# Operating an eHealth System for Prehospital and Emergency Health Care Support in Light of Covid-19

**DOI:** 10.3389/fdgth.2021.654234

**Published:** 2021-08-24

**Authors:** Efthyvoulos Kyriacou, Zinonas Antoniou, George Hadjichristofi, Prokopios Fragkos, Chris Kronis, Theodosis Theodosiou, Riana Constantinou

**Affiliations:** ^1^Department of Electrical and Computer Engineering and Informatics, Cyprus University of Technology, Limassol, Cyprus; ^2^eHealth Lab, Department of Computer Science, University of Cyprus, Nicosia, Cyprus; ^3^Department of Computer Science and Engineering, European University Cyprus, Nicosia, Cyprus; ^4^eHealth Lab, Department of Electrical and Computer Engineering and Informatics, Frederick University, Limassol, Cyprus; ^5^Ambulance Department, State Health Services Organization, Ministry of Health, Nicosia, Cyprus

**Keywords:** eEmergency, emergency medical systems, Covid-19 prehospital patient management, security, emergency dispatch

## Abstract

**Introduction:** The support of prehospital and emergency call handling and the impact of Covid-19 is discussed throughout this study. The initial purpose was to create an electronic system (eEmergency system) in order to support, improve, and help the procedure of handling emergency calls. This system was expanded to facilitate needed operation changes for Covid-19.

**Materials and Methods:** An effort to reform the procedures followed for emergency call handling and Ambulance dispatch started on the Island of Cyprus in 2016; along that direction, a central call centre was created. The electronic system presented in this work was designed for this call centre and the new organization of the ambulance services. The main features are the support for ambulance fleet handling, the support for emergency call evaluation and triage procedure, and the improvement of communication between the call centre and the ambulance vehicles. This system started regular operation at the end of 2018. One year later, when Covid-19 period started, we expanded it with the addition of several new features in order to support the handling of patients infected with the new virus.

**Results:** This system has handled 112,414 cases during the last 25 months out of which 4,254 were Covid-19 cases. These cases include the transfer of patients from their house to the reference hospital, or the transfer of critical patients from the reference hospital to another hospital with an intensive care unit or transfer of patients from one hospital to another one for other reasons, like the number of admissions.

**Conclusion:** The main purpose of this study was to create an electronic system (eEmergency system) in order to support, improve, and help the procedure of handling emergency calls. The main components and the architecture of this system are outlined in this paper. This system is being successfully used for 25 months and has been a useful tool from the beginning of the pandemic period of Covid-19.

## Introduction

The effective and quick handling of an emergency incident can be crucial for a patient's survival and recovery. This handling is directly affected by the work of first responders (paramedics), the effective dispatch procedures of ambulance vehicles, as well as the type and content of data interchanged between an ambulance vehicle, the control center, and the hospital emergency department (1, 2).

Recent advances in electronic health systems, along with technology evolutions and research in computer science can significantly help the aforementioned issues (2, 3). Despite the advances, several logistical problems appeared in emergencies due to failures to coordinate their distribution (1). To address these issues, several emergency dispatch centers around the world are increasingly using several forms of emergency dispatch protocols when handling emergency calls (2, 3). The main objective behind these protocols is to ensure that an incident is appropriately evaluated and responded. In addition, there has been a consistent effort to implement several priority dispatch protocols through computer-based systems in an effort to automate processes and further minimize human error rates (1, 4–8).

The aim of this work is to present the design of Emergency Response protocols and the development and implementation of a secure emergency handling platform that was created to support the Ambulance Department of the Ministry of Health of Cyprus. The Ambulance Department was recently restructured. The main purpose of this work was to reform and optimize the procedures of emergency call handling and Ambulance dispatch. The work started in early 2016 with the creation of a central call center and additional ambulance base stations. The call center initially started working for one region and eventually covered the entire island. All the procedures, starting from emergency dispatch to incident handling, have been organized using specific protocols. Initial work for this system was presented in a conference paper by Kyriacou et al. (9). The appearance of the Coronavirus disease (COVID-19) and the spread of the virus in Cyprus was an extra challenge to the system, which acted as a catalyst for optimizing handling of incidents and the support from high tech solutions. We present the architecture of the system; outline the user functions and system operations from the perspective of usability, efficiency and security; and we discuss the impact of Covid-19 on the operation of the system.

## Methodology

The presented system has been designed and developed according to the needs of the ambulance services so as to support the workflow followed while receiving an emergency call. This includes triage, ambulance dispatch and incident handling from paramedics (3). In extensive user requirements procedure was initially completed before the design and development phase of the system. Despite the initial definition of user requirements, the development team followed an incremental development model that was easily modified especially since there was a small group of easily accessible users. During the development phase of the system, the procedures were modified dynamically to match the reformation of the call center and ambulance services. Initially, the users and use cases were identified as described in section Use Cases.

Our proposed platform securely handles and stores Electronic Health Records. Operations were structured according to the European Union laws and directives (10).

A series of actors are involved in the use case scenarios of our system. Each actor has a specific role and executes different functions within the system. More specifically, system users have seven different types of roles: (1) Administrator, (2) Call center manager, (3) Chief of Ambulances, (4) Dispatcher, (5) Covid-19 Consultant (6) Paramedic, and (7) Doctor. The Administrator is responsible for the overall administration of the system and deals with aspects such as setting access controls and adding removing any of the other type of users. The Call Center Manager is responsible for the overall management of the activities related to the call center. He takes important decisions regarding call center and manages the Dispatchers. The Dispatchers are the personnel of the call center handling the calls and assigning Ambulances to incidents. The Chief of Ambulance handles the functions of the Ambulance unit and the Paramedics within. The paramedics are responsible for driving to the incident during an emergency event and handling the patient. The Doctors offer prehospital health care by going over submitted patient information and guiding the paramedics to treat the patient in transit.

### Use Cases

In the approach we followed, we divided the system into two main parts. The first part refers to call handling and ambulance management in the call center, while the second part refers to any activities that take place between prehospital actors after ambulance dispatch.

**Figure 1:**
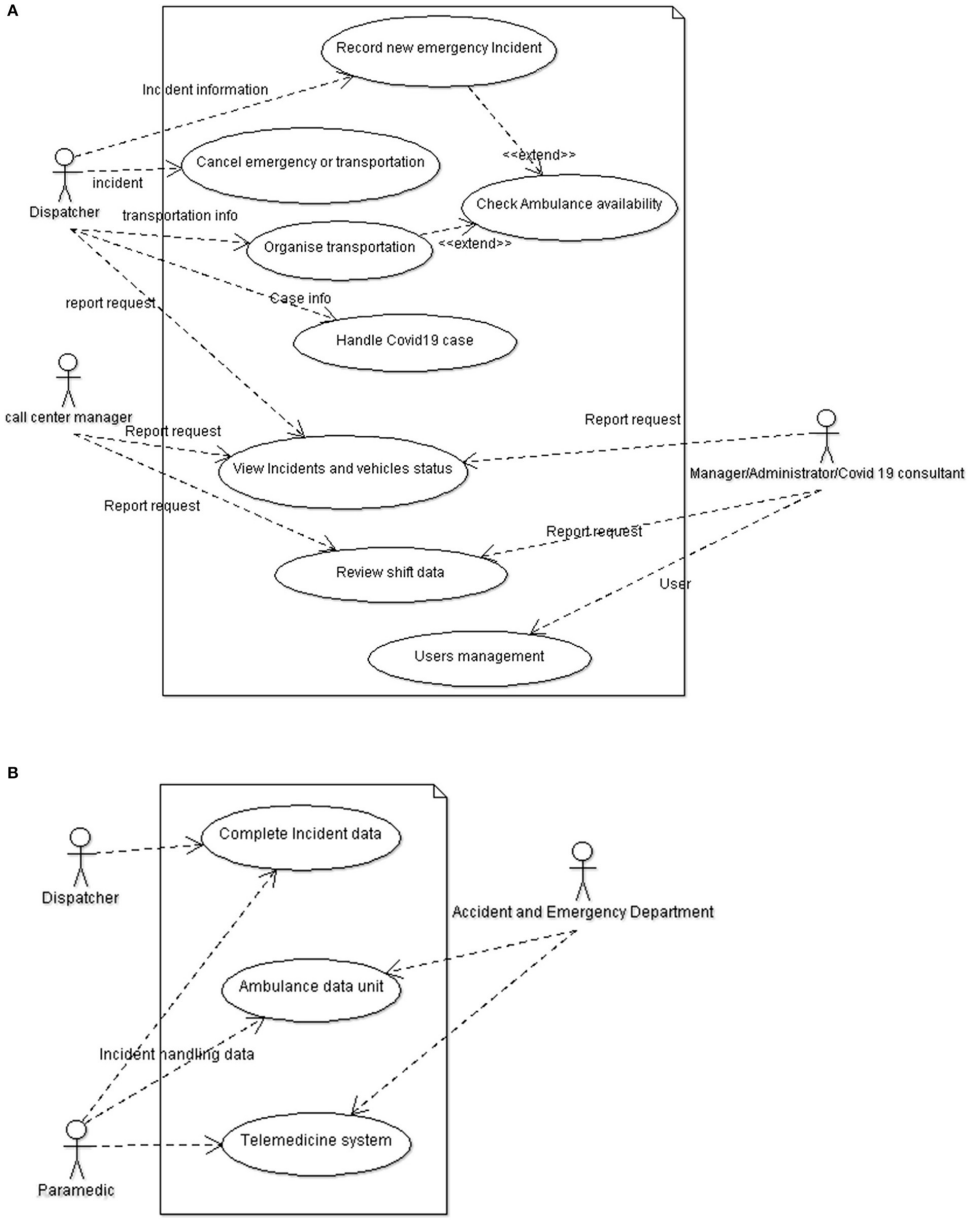
Use case diagram displaying the use cases or functions of users that take place in the system. **(A)** Part one shows the functions of users within the developed system for emergency call handling and ambulance management prior to the dispatchment of the ambulance. **(B)** Part two shows the interactions of the Dispatcher, Paramedic, and Accident and Emergency Department unit that includes doctors after the ambulance is dispatched. Through these interactions data is exchanged between call center (*via* Dispatchers), the ambulance vehicles (*via* Paramedics), and Accident and Emergency Department unit (*via* doctors).

As shown in Figure 1A, part one affects the procedures followed by the call center. The actors involved in this case are the dispatchers (trained paramedics that answer and handle the emergency call), the call center manager, the manager/administrator of the unit (ambulance services), and the Covid-19 consultant. The main use cases of this part are:

- Record a new emergency incident: This is the case when a dispatcher receives a new call. This case starts with initial evaluation of the incident based on the protocols of the center, which include triage and data recording. The availability of an ambulance is found, and the call is transferred (send an ambulance to the incident). Dispatchers are the actors involved in these cases.
- Organize transportation of a patient: Transportation needs include moving a patient from a hospital to another hospital, from their house to a hospital etc. In general, these transportations are not accident and emergency incidents related.
- Cancel emergency or transportation: An emergency or transportation is canceled for some reason and the allocated vehicle is then released.
- Handle Covid-19 cases: This scenario is the new addition to the system to handle Covid-19 incidents. These calls will be either emergency calls for transferring a patient to a hospital, or transportation calls for transferring patients from a hospital to another hospital. These calls are handled using a different protocol for staff protective measures, and urgency of transportations compared to the typical cases.
- Reporting cases: The remaining three use cases described in Figure 1A (view incidents and vehicle status, review shift data, and user's management) are managerial cases where the call center manager and the manager of the ambulance services are involved.

Part two (see Figure 1B) affects the procedure of data exchange. The actors involved in this case are the emergency call center, the ambulance system, and the accidents and emergency department. The main use cases are:

- Real-time monitoring of the patients involved in an incident scene, through a portable telemedicine unit that transmits bio-signals. This allows on-call doctors at the First aid control center to make decisions a-priori for in hospital patient handling (e.g., support stroke or cardiac clinic decision for patients needing emergency angioplasty).
- Get initial call data from the call center and complete any data related to the transportation and handling of the patient by the ambulance paramedics.
- Get data related to the handling of Covid-19 patients and provide any needed guidance and support for patient handling.

### Emergency Call Workflow Description

**Figure 2:**
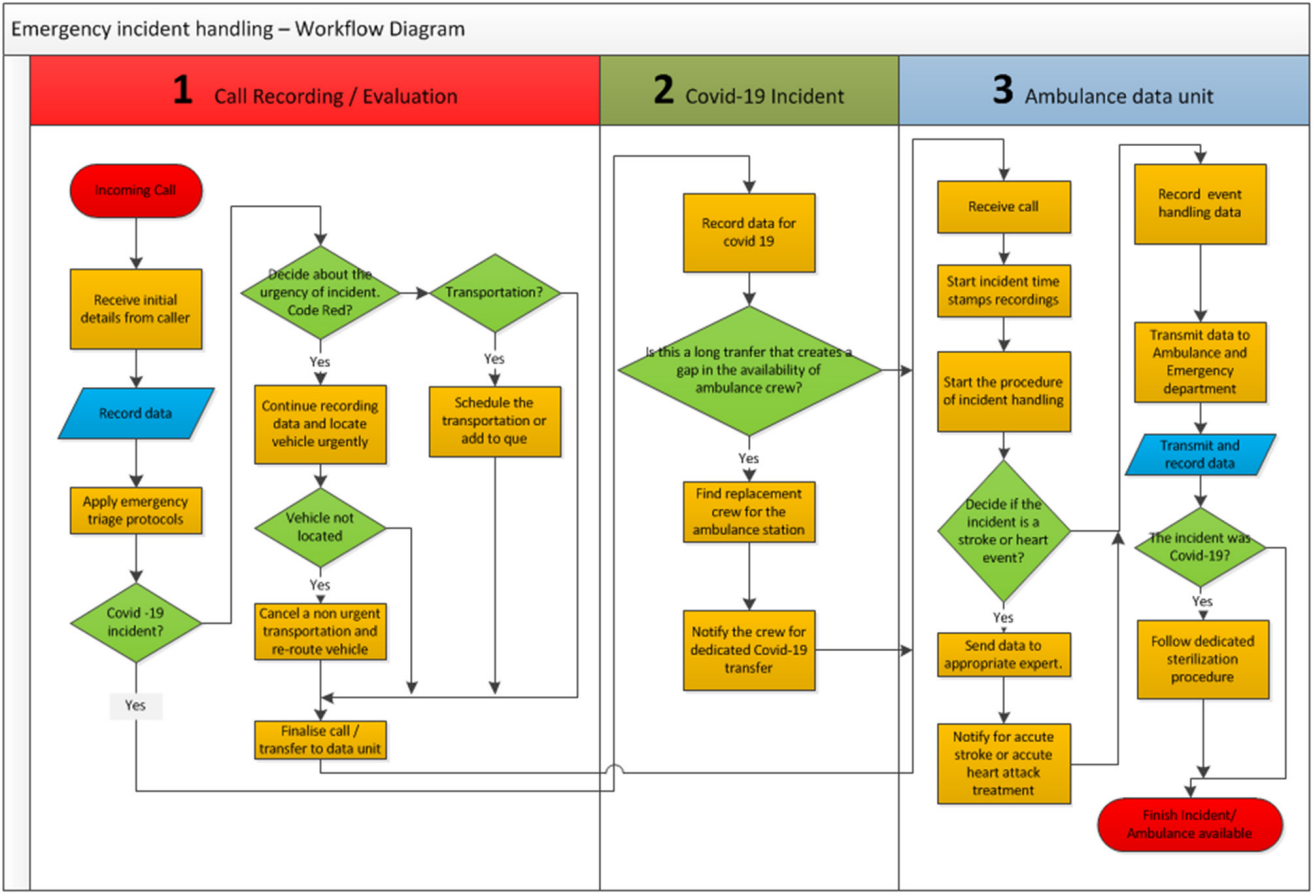
Emergency procedures—workflow management system.

Emergency calls are handled based on a specific set of steps. These steps have been edited to incorporate the actions of a Covid-19 consultant. The workflow of actions that take place during an emergency call scenario is described as follows (see Figure 2):

- An emergency call starts at the call center.
- The dispatcher receives input from the caller and follows the triage protocol in order to assess the incident.
- The dispatcher records initial information about the incident, including Covid-19 related information, and if possible record basic information about the patients involved. If the patient is a possible Covid-19 or a verified case, the paramedics are informed in order to take the appropriate protective measures and follow the designated protocols for Covid-19.
- The dispatcher chooses an available ambulance and paramedics and dispatches them to the location of the incident.
- Information recorded is transmitted to the Ambulance Data Unit.
- Following the emergency dispatch procedure, paramedics arrive at the incident location, assess the patient's condition, and inform the Ambulance Dispatching Unit about the status of the patient/incident.
- If needed, they also transmit bio-signal information to the hospital so that the doctor on call can view, assess the severity of the situation, and guide the paramedics accordingly. Once the patient is transferred to the hospital, the data regarding the incident are also transferred to the data station of the accidents and emergency department.
- Soon after the delivery, the paramedics have to take all the necessary procedures for cleaning and sterilization of the vehicle. Then they inform the call center that the ambulance is available to service other requests. If the ambulance is carrying a Covid-19 patient, it is sterilized based on a Covid-19 specific protocol and then released to other service requests.

### System Architecture

The system architecture is mainly divided into two major components. The first component is the system responsible for managing the incident call, ambulance vehicles, and initial information recorded, it covers part 1 of the user requirements. The second component covers the ambulance vehicle part which includes data exchange processes between ambulance vehicles, call center and hospitals. This refers to part 2 of the user requirements. This gives the ability to exchange data like incident initial description, incident handling procedure, and bio-signals of a patient, between the ambulance vehicles, the call center and the referring hospital/doctor.

**Figure 3:**
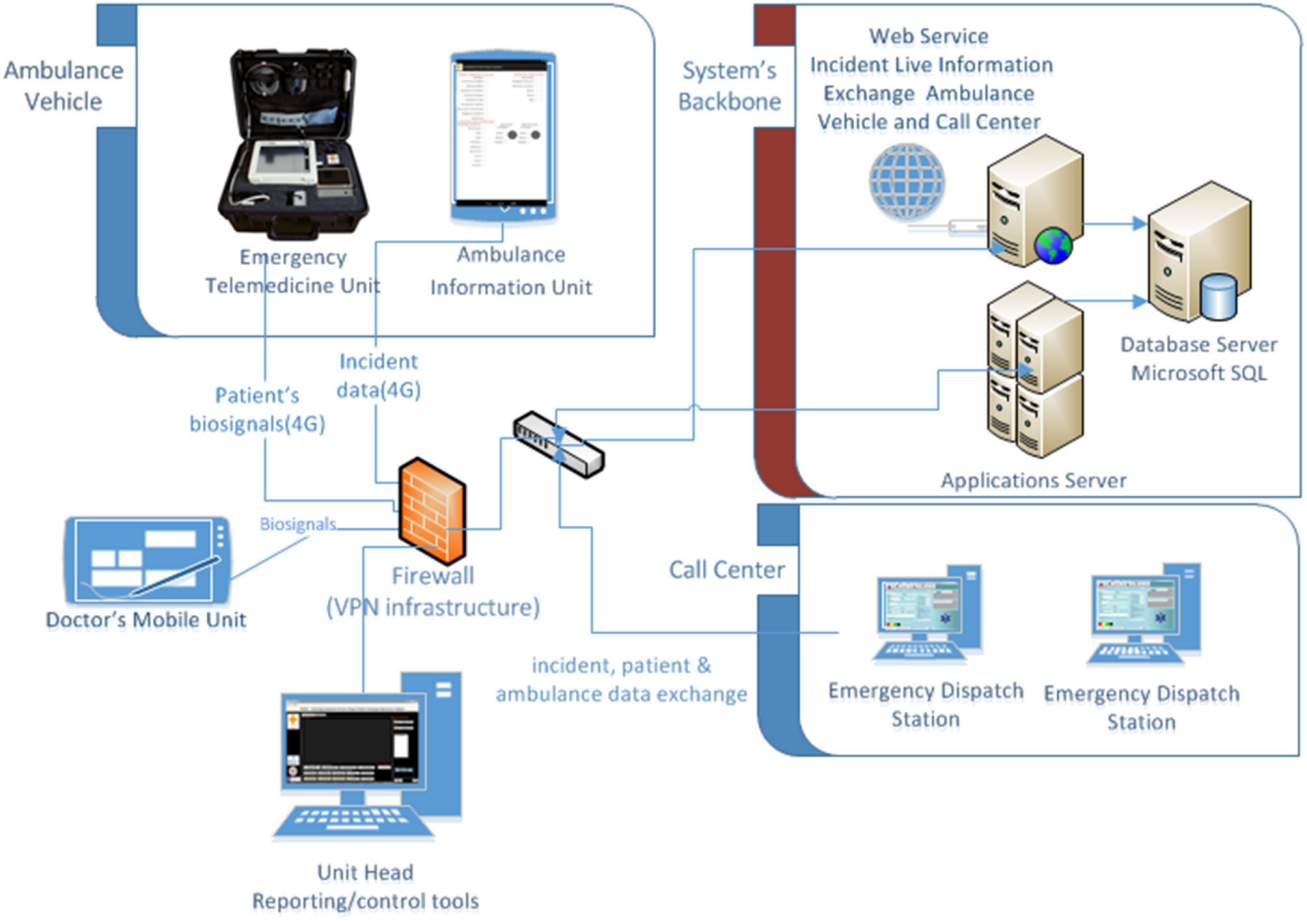
eEmergency health care system architecture.

Based on the aforementioned operations and user requirements we designed the system as shown in Figure 3. The procedures followed by the ambulance department are based on specific protocols that can differ for each case. These protocols were created by the Ambulance Department according to internationally accepted protocols and standards published from associations like NAEMT- National Association of Emergency Medical Technicians—https://www.naemt.org/. All these protocols are supported by the system. The detailed workflow of incident handling is depicted in Figure 2 and described in section Emergency call Workflow description.

The recorded data can be made available to the accident and emergency department of a main hospital (the place where the ambulance transfers the patient), to the call center and to the ambulance vehicle. The ambulance vehicles have the technology to exchange data and vital bio signals from the scene and during transportation of a patient (11) using 4G wireless technology.

As can be seen in Figure 3, the ambulance vehicle is equipped with an Ambulance Data Unit and an Emergency Telemedicine Unit. These units are the evolution of the work presented in (11). The Emergency Telemedicine Unit is responsible for sending bio-signals (ECG 3-7 leads, spO_2_, blood pressure, temperature, and respiration). A doctor on call can then use the Doctor's Mobile Unit (e.g., a tablet or pc), authenticate and get authorized into the server, and monitor a patient's condition.

The Ambulance Data Units are Android tablets equipped with 4G communication cards. The paramedics use the tablet (or pc) to get initial information about the incident (recorded during the emergency and the triage procedure from the call center staff) and concurrently report information regarding the incident. These include information about incident handling and procedures followed by the ambulance paramedics. Additionally, the information can include patient, or incident info. All this information together with information related to device and user authentication are hosted on the servers that act as a backbone of the system as shown in Figure 3.

The communication between the mobile units, the call center and the hospitals is feasible through TCP/IP protocols over mobile 4G wireless networks. Data from ambulances to the server are exchanged using web services. Access control to the system is enabled using a unique ID for each unit, Secure Socket Layer (SSL) for communication, Virtual Private Network (VPN) infrastructure through a firewall, and different access levels depending on the user group (see section Security Design).

System data storage is supported by a dedicated database server. The entities include system users, where each group of users has different access levels. The database system has the ambulance entity, where each vehicle and its status are recorded, the patient entity which stores general information about a patient, the incident entity, which stores any information regarding the incident (medication, handling procedure, initial diagnosis, final diagnosis, etc.), the bio-signals entity, which stores any real time bio-signals transmitted during an incident, and the logs entity which stores the activity done and the user that did it.

The main aspects of system architecture are described in the following section Security Design up to Ambulance Data Units.

#### Security Design

Based on the scenarios described, the system supports users having seven different types of roles: (1) Administrator, (2) Call center manager, (3) Chief of Ambulances, (4) Dispatcher, (5) Covid-19 Consultant, (6) Paramedic, and (7) Doctor. Each role requires different access controls.

Access control takes into account the user identity, as well as the identity of the connected devices. The Administrator has the highest authority in the system. He is responsible for registering and authorizing the Chief of Ambulances (shown as call center manager in Figure 1A), the Shift Managers, and the Dispatchers. They are each given a unique username and password and are authorized to handle various operations relevant to the Ambulance Department in which they belong. This authentication material is managed offline by the system administrator.

Doctors using the Doctor Mobile Unit are considered as external entities. Authentication is executed both on the user's ID and the device's ID connected to the system. This setup enables multiple users to use the same Doctor Mobile Units. Further, a Doctor Mobile Unit is tied to a specific location and thus once this device is authenticated it allows for authorization of specific operations within the system. Following an incident, the authentication of Doctors' ID on the Doctor Mobile Unit authorizes them to get access only to their assigned incidents. Doctor Mobile Units can be Android devices, which have a unique ID.

The Ambulance Data Units used by paramedics utilize double authentication. Thus, during an emergency both the user and the unit are identified and authorized for access. Introducing per user authentication in addition to per unit authentication, prevents someone stealing the device from altering information regarding an incident or submitting wrong information. Currently, there are no physical security controls to prevent anyone from stealing the Ambulance Information Unit. One such control that can be introduced in the future is the installation of a small safe deposit box with a digital key within the ambulances.

Each ambulance unit is assigned a specific Android device and is used by different groups of paramedics depending on the scheduled shifts. Within each group, an individual is granted access and assigned the responsibility for data entry. In this manner, the system holds accountable a specific individual that worked during a specific shift for filling up incident-related information. A strength of this setup is that only one individual needs to be trained to handle the device. A weakness, however, is that in case that individual needs to be replaced or is absent, it creates complexities in terms of finding a trustworthy substitution.

Duration of access to the system is controlled through session keys. Session keys are dynamically generated using hash functions after a user gets authenticated into the system. The validity period of a session key depends on the length of time that the user needs to access the system and the criticality of the operation. Frequent refreshing would imply a more critical operation.

A summary of the various access levels in the system per user type is offered in Table 1. The Administrator, the Chief of the Ambulance Services and the Shift Manager have the most important role in the system. Separation of duties is utilized, and different tasks are assigned to these two users. The Administrator is responsible for overseeing that the network operations are being carried out effectively and the system is always available. He/she registers any type of users including the Chief of Ambulance Services, the Shift Manager, the Covid-19 Consultant, Dispatchers, Doctors, and Paramedics. However, the Chief of Ambulance and the Shift Managers have the general responsibility of overseeing the correct operation of the Ambulance Department. They can also add or remove new ambulances joining the system. Similar to the Administrator, they may add dispatchers, for the scenario where the Administrator is unavailable. In addition, the Chief of Ambulance is the only one who can add or remove Shift Managers. Dispatchers can create a new incident, update an existing incident, but cannot delete any incident for any reason. They can view the ambulance entity and alter the status of an ambulance based on the emergency schedule. The status of the ambulance could be “On Call,” “Available,” or “In Service.” Dispatchers can add or modify the records of new patients and link the patient to a newly recorded incident. Furthermore, the dispatchers should be able to view/edit all incidents recorded by their co-workers at the Ambulance Emergency Call Center. The dispatchers should also be able to see information that is being sent by Ambulance crew at real time regarding the incident status and alter the status of an ambulance in the current shift.

**Table 1:** Role based access control to the different modules of the system.

	**Role Roles of participants in the emergency unit**						
**Database Entities**	Administrator	Shift manager	Chief of ambulances	Dispatcher	Covid-19 Consultant	Paramedic	Doctor
User	R/W(A,L,M)	R/W(A,L,M)^***^	R/W(A,L,M)^**^	**-**	**-**	**-**	**-**
Ambulance	**-**	R/W(A,L,M)	R/W(A,L,M)	W(M)^****^	R/W(Ap)	W(M)^****^	**-**
Patient	**-**	R	R	R/W(A,M)	R/W(Ap)	R/W(A,M)	R/W(Ap)
Incident	**-**	R	R	R/W(A,M)	R/W(Ap)	R/W(M)	R^*^
Biomedical signals	-	-	-	-	-	W(A)	R
Logs	R	R	R	-	R/Ap		-

*R, read; W, write; -, no permission.*

*W(A, add; L, Lock; M, Modify; Ap, append).*

* *For Incident tied to patient.*

** *For adding Dispatchers and Shift Managers only.*

*** *For adding Dispatchers only.*

**** *Modify the status of the Ambulance (available, on Call, In Service).*

*Modify implies Append, unless Append is stated instead*.

The role of the Covid-19 consultant is to provide guidance to the Ambulance Center Personnel regarding all aspects of its operations as impacted by Covid-19. Thus, the access control of the consultant is restricted to mainly append on records of ambulances, patients, incidents, and logs. This access enables consultants to introduce comments, such as the need for certain personnel to get tested for Covid-19, or the need for certain procedures to be altered or followed. Furthermore, it allows them to keep track of all the incidents handled and provide recommendations on how future incidents need to be handled.

Doctors can only read and append to patients' records. They also have read access for the incidents in which their patient was involved. Paramedics can add a patient to the system or modify a patient's records. They can also update the status of the patient's incident.

A logging mechanism is used throughout the entire system to track the events so as to provide accountability. Every instance of our database has a log file associated with it, which is accessible for reading only by the Chief of Ambulance, the Covid-19 consultant and the Administrator. Log files include incident_log, ambulance_log, patient_log, and logs capturing the activity of users, such as the doctor_log, the paramedic_log, and the dispatcher_log. Typically, the Chief of Ambulance will look at the logs to investigate issues regarding the operation of the Ambulance Unit, such as the effectiveness of an operation. On the other hand, the Administrator may look at the logs to investigate any malicious or erroneous behavior of the users in the system. In light of Covid-19 the Covid-19 consultant uses the log to keep track of Coronavirus incidents and assess the effectiveness of handling events. Items, which might be of interest are the number of Coronavirus patients per location, the severity of the various cases, and the methodology of treatment. In order to promote safety, the personnel and ambulances involved per Covid-19 incidents is also accounted for. Paramedics handling Covid-19 incidents are frequently checked for a possible infection and where possible are prevented from participating in non-corona virus related incidents. Thus, a separation of responsibilities is established based on the type and severity of incident handled. Lastly, the sanitization schedule of the ambulances is set up based on the number and frequency of incidents.

Note that nobody in the system has access to delete any records. An option would be to start deleting records after a certain number of years. However, that period would depend on the policies adopted by the Ambulance Department.

In addition to access control, another important security property is privacy. Privacy is tied to the confidentiality of data, which is achieved through the encryption of transmitted data. All external entities connecting to the system, such as the ambulance data units, utilize the FortiClient VPN by Fortinet (12) with SSL VPN activated to provide privacy. Confidentiality is even more critical with Covid-19, as it is important to protect the Identity of infected people and their location. Note that encryption is not used in the Communication between the bio-signal server and the Emergency Telemedicine Unit. The induced encryption overhead would be operation prohibitive.

#### Data Exchange

One of biggest challenges in this system was the accurate exchange of medical data and availability of data to all the parties involved (call center, ambulance vehicles, reference hospital/doctor). Toward this direction, there was a great need to enable the seamless tablet devices' communication with the emergency handling platform by implementing the System Communication module as shown in Figure 3. This facilitates real-time monitoring of the patients involved in an incident scene during his/her transportation from the incident scene to the hospital. Thus, it allows the Emergency Department healthcare providers' to be well-prepared for the treatment of the patient as soon as he/she arrives at the hospital premises.

The Emergency handling platform was installed in two-tier client-server architecture in which the functional process logic, data access, user interface, and computer data storage were developed and maintained as independent modules on two separate virtual servers (see Figure 3). Both virtual servers are located on a physical server located at the Ambulance Department premises. This is installed in a dedicated server room with all the appropriate security measures, data backup devices, and power failure support devices.

The first tier corresponds to the Application and Presentation Layer. This layer uses an application server that contains the functional business logic of the system, which drives an application's core capabilities and the user interface. The communication with the specific Android application that is installed on a tablet device is enabled through Application Programming Interface (API) calls using Representational State Transfer (REST) API. API is a set of clearly defined methods of communication among various components. It is a predefined set of rules that allow programs to talk to each other. We created the APIs on the server and verified that the client android application can talk to it. REST determines how an API looks like. REST is a set of rules that we followed when we were creating our APIs. One of these rules states that the android application is able to get a piece of data (called a resource) when it links to a specific URL. Each URL is called “a request” that triggers an operation on the server while the data sent back to the android application client is called “a response.”

The second tier corresponds to the Data Layer. This specific layer handles a Microsoft SQL database management system that provides secured access to application data. Data is communicated through SQL stored procedures. A stored procedure is a prepared SQL query that is stored on the SQL server so that it can be reused repeatedly by just calling and executing the stored procedure. Stored procedures enhance security of the system and minimize injections by ensuring that operations being performed are allowed by the user. That means that *ad hoc* queries and data modifications can be disallowed. This prevents users from maliciously or inadvertently destroying data or executing queries that impair performance on the server or the network. Furthermore, stored procedures can reduce network traffic by combining multiple operations into one procedure call.

The system supports auditing mechanism by performing security audits on every API call using audit trails and logs that offer a back-end view of system usage. Audit trails and logs record key activities, showing system threads of access, modifications, and transactions.

Data is accessed on the Ambulance Data Units (Android app)via secure API calls using in JavaScript Object Notation (JSON) format that are implemented in ASP.NET user-interface (view), data (model), and application logic (controller) (MVC) environment. Once a paramedic user opens the android application, he/she must provide valid credentials in order to be authorized and authenticated by the system to access data. The System administrator is responsible to create and provide valid credentials to paramedics.

**Figure 4:**
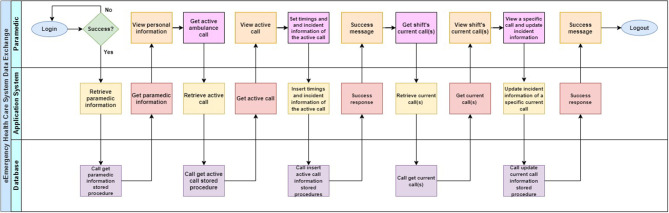
eEmergency health care system data exchange swim lane diagram.

API calls support the following functions so as to facilitate the communication with the app: (a) login, (b) get paramedic information, (c) get active ambulance call, (d) insert information regarding the patient(s) of an active call, (e) get ambulance calls that were performed during the paramedic's shift, (f) update call information, and (g) send SOS alert. A related data exchange flow that depicts interaction between the user and the two-tier client-server architecture using REST APIs can be seen in the corresponding swim lane diagram in Figure 4.

The system allows a paramedic to update information about an ambulance call that was performed the last 6/12 h, based on the corresponding shift duration, to update any information or add missing information. Due to legal issues that might be raised, ambulance response datetime and ambulance station departure datetime fields, once recorded they cannot be updated. Moreover, the system allows a paramedic to send an SOS alert to the Ambulance call center directly from the app to call for help (this can be used in cases where the paramedics face a thread from a patient or other people located at the incident scene).

#### Ambulance Data Units

Ambulance data units are assigned specific Android devices (Lenovo Tab E10) which are designated to ambulance vehicles depending on the scheduled shifts. Each Android device functions as an intermediary between the paramedics/ambulance and the server responsible for data exchange. The application running on each device is designed to record and update information of incidents recorded for the first 6/12 h. Once 6/12 h have passed since the creation of the incident, the information gets hidden on the device, and thus no modification is allowed. Tablet access is restricted only on the application for data exchange, this secures any unwanted access to internet data or applications which can cause serious problems to our system.

As depicted in Figure 2 in the Ambulance Data Unit (section Results and Discussion), the application is divided into various activities, which indicate when the fundamental transactions occur between the user and the system. Once a connection to the server has been established, the user is prompted to enter his/hers credentials. The system will then authenticate the user.

Once authenticated, the user is presented with different information according to their role (generic or not generic). Generic users in the system exist for administrative use only, whereas non-generic users are regular users, which are disparate entities within the system. Before accessing incident data, non-generic users will be prompted to enter their assigned station and shift period. For generic users, their ID and ambulance number is also required.

**Figure 5:**
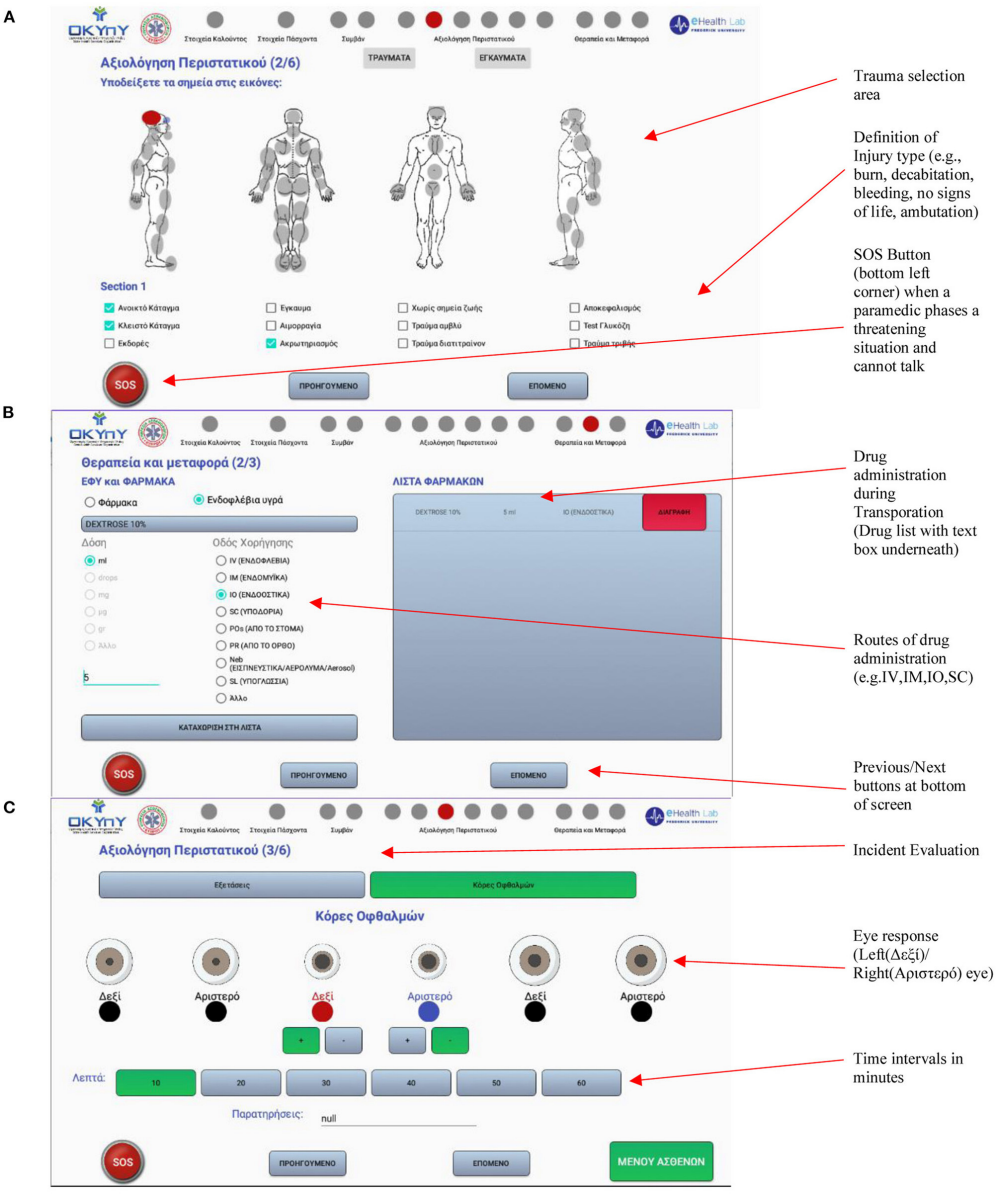
Some of the key screenshots of the GUI used to record incident data **(A)**. Incident Evaluation for traumas **(B)**. Medicine administered during transportation **(C)**. Incident evaluation based on for eye response.

Incident data is represented in the form of a table and is retrieved every time the user visits the incident selection screen. Based on the date of creation, ambulance station name and patients, users are able to recognize the incident of their choice. Once an incident is selected, the user can navigate the menu responsible for handling the records for each patient (see Figure 5). Each incident is composed of patient incident records, which can either be created or updated by the users. Through the ambulance data unit, paramedics are able to record all data regarding the incident. Screens used for recording of trauma, drug administration and eye response evaluation are shown in Figure 5. Deletion is possible only for records, which have been stored statically/temporarily in the application, and thus do not coincide with the contents of the database. If a patient record has already been stored within the incident, it can be deleted temporarily. The deleted data can be retrieved upon reentering the selected incident.

Each patient incident record contains sensitive data, which is sent in the form of a status report to a previously designated email address. Users may also choose to send the report to more email addresses, each corresponding to an ambulance unit. Completing such information requires the utmost attention from the users.

## Results and Discussion

The presented system has been completed and has been in daily practice routine for about 25 months. The system was adopted for daily practice after using it for 5 months as a test phase. During these 5 months, the system was thoroughly tested, and any errors were corrected. Additionally any minor additions requested by the users were added.

Each incident in the system is tagged with various priority levels, which are denoted with color codes. The color codes are Red (Immediate response extremely urgent case), Yellow (case with serious problems personnel can response up to 20 min), Purple (denotes transportation of patient. This can be a prescheduled transportation or a transportation from one hospital to another), Green (case which can wait up to 3 h) and black (meaning the person is dead). The number of incidents per priority level can be seen in Table 2.

**Table 2:** Total number of recorded incidents from 1st December 2018 until 14 January 2021.

**Priority level**	**Phase 1 (test)**	**%Phase 1**	**Phase 2**	**%Phase 2**	**Phase 3 (Covid-19 first wave)**	**%Phase 3**	**Phase 4 (Covid-19 second wave)**	**%Phase 4**	**Total**
Red	6,739	29%	14,584	31%	5,086	27%	5,423	23%	31,832
Yellow	9,739	42%	19,713	42%	8,561	45%	10,980	47%	48,993
Purple	6,237	27%	11,169	24%	4,923	26%	6,281	27%	28,610
Green	481	2%	771	2%	400	2%	449	2%	2,101
Black	190	1%	337	1%	179	1%	172	1%	878
**Total**	**23,386**		**46,574**		**19,149**		**23,305**		**112,414**

*The division of the incidents is based on priority levels. Bold values emphasize the total values*.

The test phase 1 was between 1st December 2018 until 30th April 2019. During this phase, the system recorded 23,386 cases out of which 6,739 cases (29% of total cases) were code red cases as shown in Table 2. Soon after the test phase 1 the system entered the regular use in daily practice. This is shown as phase 2. It started from May 1st 2019 until the 29th of February 2020. This new timestamp was added when Covid-19 incidents started appearing in Cyprus. The number of incidents recorded during phase 2 from the system were 46,574 out of which 14,584 were code red (31% of total cases).

Soon after covid-19 cases started appearing in Cyprus (around the beginning of March 2020). The system was dynamically adopted in order to cover the new cases. This period started from March 1st 2020 until now January 14th 2021 (when this paper was submitted).The pandemic Covid-19 period is also divided into two periods. The first wave of the pandemic starting from March 1st 2020 until July 31st 2020 and the second wave starting from August 1st 2020 until the 14th of January 2020.

Comparing the different periods of observations it can be seen that the percentages of priority levels of each phase are similar except from the red and yellow codes. Red was reduced while yellow was increased during the covid-19 and the lockdown periods that took place. This is something observed through systems of other countries also as shown in (13). Red incidents still have a high percentage because of incidents like stroke and heart attacks also shown in (14). Trauma cases (red code) were reduced which explains the small reduction of this group. The increase in yellow codes was expected as covid-19 calls increased (these calls need special attention but are not at the urgency of level red).

Since March 2020, when the pandemic period started in Cyprus, 4,152 emergency incidents regarding Covid-19 cases have been recorded. The Covid-19 cases have been continuously increasing because of the increased incidents recorded during the second wave of the pandemic, which proved to be much tougher to handle than the first wave. Based on the statistics published by the government of Cyprus available on the page of the World Health Organization (WHO) (15), the total cases reported are 28,124 in total until January 14th 2021. In detail, the total cases compared to the total emergency incidents and covid-19 emergency calls are shown in Table 3.

**Table 3:** Recorded incidents during Covid-19 period, starting from March 1st 2020 until January 14th 2021.

	**Covid-19 first wave**					**Covid-19 second wave**						
	**March** **2020**	**April** **2020**	**May** **2020**	**June** **2020**	**July** **2020**	**August** **2020**	**September** **2020**	**October** **2020**	**November** **2020**	**December** **2020**	**January** **2020**	**Total**
Covid-19 cases	230	613	100	53	88	403	256	2,474	6166	11,636	6,105	28,124
Emergency calls	3,739	3,174	4,061	3,823	4,352	4,202	4,285	3,990	3,882	4,767	2,179	42,454
Covid-19 calls	312	294	201	72	89	192	153	552	705	1,317	582	4,469
Percentage of Covid-19	8.3%	9.3%	4.9%	1.9%	2.0%	4.6%	3.6%	13.8%	18.2%	27.6%	26.7%	10.5%

Handling of covid-19 cases created the need for communication and synchronization of the Ambulance Department database and the Cyprus' National Contact Point eHealth (NCPeH) database. NCPeH enables seamless cross-border care and secure access to patient health information between European healthcare systems (16). This will correlate the Patient Summary record of a given patient to be updated automatically when a patient that is a verified Covid-19 incident will be transferred by an ambulance vehicle. Additionally, the Patient Summary will be updated accordingly to include all the corresponding data of that patient, including bio-signals, current medication, active problems and other medical information that will be recorded by the paramedic using the Ambulance data units.

## Conclusion

The presented system has been successfully used for around 25 months without any major technical problems. One of the main challenges of the system was to follow the restructuring of operation procedures of the Ambulance Department. The restructure caused a lot of changes such as the creation of a central call center, the creation of extra ambulance stations, introduction of newly established protocols and others.

The design and implementation of the new digital system was a challenge for our group. We had to follow the restructure procedure and adapt according to the changes. The general aim was to support the Ambulance Department of the Ministry of Health in Cyprus. The key contribution of this work is the design of an integrated system that supports emergencies by allowing the dynamic assignment of paramedics and ambulances to incidents during an emergency and monitoring the procedure from the moment a call is received until a patient is delivered to a hospital. The main task is to minimize mistakes and create a more effective emergency handling system.

Appearance of Covid-19 and the start of the pandemic period was another challenge that we had to phase with our ehealth system. The stress on the emergency health care system was high and all these procedures needed accurate and immediate data exchange. This was also supported with success.

## Data Availability Statement

The datasets presented in this article are not readily available because they contain classified medical data. Requests to access the datasets should be directed to efthyvoulos.kyriacou@cut.ac.cy.

## Author Contributions

EK: system concept, system development, and project management. ZA: system restructure, web service development for ambulance, and mobile devices communication. GH: system security. PF: ambulance data unit and communication module development. CK: call centre core system development. RC: system concept, system user requirements, and system evaluation. TT: system user requirements and system evaluation. All authors contributed to the article and approved the submitted version.

## Conflict of Interest

The authors declare that the research was conducted in the absence of any commercial or financial relationships that could be construed as a potential conflict of interest.

## Publisher's Note

All claims expressed in this article are solely those of the authors and do not necessarily represent those of their affiliated organizations, or those of the publisher, the editors and the reviewers. Any product that may be evaluated in this article, or claim that may be made by its manufacturer, is not guaranteed or endorsed by the publisher.
